# Gene expression profiling to predict recurrence of advanced squamous cell carcinoma of the tongue: discovery and external validation

**DOI:** 10.18632/oncotarget.18692

**Published:** 2017-06-27

**Authors:** Tomohiro Enokida, Satoshi Fujii, Mari Takahashi, Youichi Higuchi, Shogo Nomura, Tetsuro Wakasugi, Tomoko Yamazaki, Ryuichi Hayashi, Atsushi Ohtsu, Makoto Tahara

**Affiliations:** ^1^ Department of Head and Neck Medical Oncology, National Cancer Center Hospital East, Kashiwa, Chiba 277-8577, Japan; ^2^ Advanced Clinical Research of Cancer, Juntendo University Graduate School of Medicine, Bunkyo-Ku, Tokyo 113-8421, Japan; ^3^ Division of Pathology, Exploratory Oncology Research & Clinical Trial Center, National Cancer Center, Kashiwa, Chiba 277-8577, Japan; ^4^ Department of Digestive Endoscopy, National Cancer Center Hospital East, Kashiwa, Chiba 277-8577, Japan; ^5^ Biostatistics Division, Center for Research Administration and Support, National Cancer Center, Kashiwa, Chiba 277-8577, Japan; ^6^ Head and Neck Surgery Division, National Cancer Center Hospital East, Kashiwa, Chiba 277-8577, Japan; ^7^ National Cancer Center Hospital East, Kashiwa, Chiba 277-8577, Japan

**Keywords:** tongue squamous cell carcinoma, oral squamous cell carcinoma, gene expression profile, keratin 4, cytokeratin 4

## Abstract

**Objectives:**

To establish a prognostic signature for locally advanced tongue squamous cell carcinoma (TSCC) patients treated with surgery.

**Results:**

In the discovery study, unsupervised hierarchical clustering analysis identified two clusters which differentiated the Kaplan-Meier curves of RFS [median RFS, 111 days vs. not reached; log-rank test, *P* = 0.023]. The 30 genes identified were combined into a dichotomous PI. In the validation cohort, classification according to the PI was associated with RFS [median RFS, 754 days vs. not reached; log-rank test, *P* = 0.026 in GSE31056] and DSS [median DSS, 540 days vs. not reached; log-rank test, *P* = 0.046 in GSE42743 and 443 days vs. not reached; *P* < 0.001 in GSE41613]. Among genes, positive immunohistochemical staining of cytokeratin 4 was associated with favorable prognostic values for RFS (hazard ratio (HR), 0.591, *P* = 0.045) and DSS (HR, 0.333, *P* = 0.004).

**Materials and methods:**

We conducted gene expression profiling of 26 clinicopathologically homogeneous advanced TSCC tissue samples using cDNA microarray as a discovery study. Candidate genes were screened using clustering analysis and univariate Cox regression analysis for relapse-free survival (RFS). These were combined into a prognostic index (PI), which was validated using three public microarray datasets of tongue and oral cancer (123 patients). Some genes identified in discovery were immunohistochemically examined for protein expression in another 127 TSCC patients.

**Conclusion:**

We identified robust molecular markers that showed significant associations with prognosis in TSCC patients. Gene expression profiling data were successfully converted to protein expression profiling data.

## INTRODUCTION

Cancer of the oral cavity is the most common malignancy of the head and neck, accounting for about 263,900 new cases and 128,000 deaths each year worldwide [1]. The most common malignancy arising from the oral cavity is squamous cell carcinoma of the tongue (TSCC). The incidence of TSCC has recently increased. Almost half of patients are initially diagnosed at an advanced clinical stage, such as c-Stage III and IV [2], and 40%-60% of all cases recur after local treatment within two years [3, 4]. Although several approaches to improving therapy have been attempted, prognosis remains poor: once a lesion recurs, median overall survival is 10 months [5]. Factors indicating a high risk of recurrence include exposure of cancer cells at the surgically resected margin of the primary tumor and extra-capsular invasion of cancer cells at a metastatic lymph node [6]. Nevertheless, accurate prediction of recurrence based on clinicopathological factors only such as delayed lymph node metastasis is difficult, which is in turn reflected in the difficulty of establishing customized therapy for patients which considers the risk of recurrence.

‘Omic’ technologies such as gene expression profiling have been used to identify subgroups of patients with a poor prognosis in other types of cancer, including breast, colon and lung cancer [7–9]. In head and neck cancer, microarray studies have provided useful biomarker candidates, especially in larynx cancer, and these have enabled the prediction of delayed lymph node metastasis. Thus, these biomarkers have the potential to be used in treatment decision making [10, 11]. Biomarkers associated with prognosis and therapeutic response require ongoing re-evaluation to ensure consistency with clinical findings. However, very few biological markers of TSCC have been identified, and their validity remains largely unknown.

Here, we used cDNA microarray to detect differentially expressed genes which could be used as biomarkers to distinguish patients with homogeneous advanced TSCC at high risk of recurrence following initial treatment of the primary tumor by surgical resection. We then confirmed these results using immunohistochemical analyses to establish protein markers associated with recurrence-free survival (RFS) and disease-specific survival (DSS), which could be immunohistochemically examined by staining.

## RESULTS

### Clinicopathological characteristics of patient cohorts in the discovery and validation studies

Clinicopathological characteristics of patients in the discovery and validation studies are shown in Table 1. No statistically significant differences between the two groups were seen except with regard to alcohol consumption, which was higher among patients in the validation study.

**Table 1:** Clinicopathologic characteristics of patients in the discovery and validation studies

Variables	Discovery study (N=26)	Validation study (Immunohistochemical staining) (N=127)	*P* value
**Age† Mean ±SD (range)**	59.80 ±13.6 (37-85)	57.03 ±14.6 (24-88)	0.819
**Gender (%)**			
Male	18 (69)	97 (77)	0.442
Female	8 (31)	30 (24)	
**Smoking status (%)**			
Never	9 (35)	32 (25)	0.323
Current/former	17 (65)	95 (75)	
**Alcohol status (%)**			
Never	10 (38)	23 (18)	0.022
Current/former	16 (62)	104 (82)	
**Surgery (%)**			
Partial resection	3 (12)	33 (26)	0.396
Hemi glossectomy	4 (15)	14 (11)	
Subtotal glossectomy	17 (65)	66 (52)	
Total glossectomy	2 (8)	14 (11)	
**Treatment form (%)**			
Surgery alone	22 (84)	112 (88)	0.623
Surgery + PORT	2 (8)	7 (5)	
Surgery + POCRT	2 (8)	8 (6)	
**pT stage (%)**			
T1-2	3 (12)	35 (28)	0.085
T3-4	23 (88)	92 (72)	
**pN stage (%)**			
N0-1	10 (38)	45 (35)	0.769
T2-3	16 (62)	82 (65)	
**UICC pStage (%)**			
Stage III	2 (8)	36 (28)	0.051
Stage IVa	24 (92)	89 (70)	
Stage IVb	0 (0)	2 (2)	
**Histological differentiation (%)**			
Well	20 (77)	81 (64)	0.197
Moderate/poor	6 (23)	46 (36)	
**Extranodal spread of lymph node metastasis (%)**			
Absent	24 (92)	96 (76)	0.059
Present	2 (8)	31 (24)	
**Positive Margin**			
Absent	25 (96)	107 (84)	0.138
Present	1 (4)	20 (16)	
**Vascular invasion (%)**			
Absent	4 (15)	24 (19)	0.673
Present	22 (85)	103 (81)	
**Lymphatic invasion (%)**			
Absent	21 (81)	101 (80)	0.866
Present	5 (19)	26 (20)	
**Perineural invasion (%)**			
Absent	15 (58)	68 (54)	0.296
Present	11 (42)	59 (46)	

NOTE: The t-test was used for continuous variable and the Pearson test or Kruskal-Wallis test for categorical variables. †Continuous variables. UICC, Union for International Cancer Control; PORT, postoperative radiotherapy; POCRT, postoperative chemoradiotherapy.

### Unsupervised hierarchical clustering of microarray data sets derived from 26 TSCC patients in the discovery study

**Figure 1:**
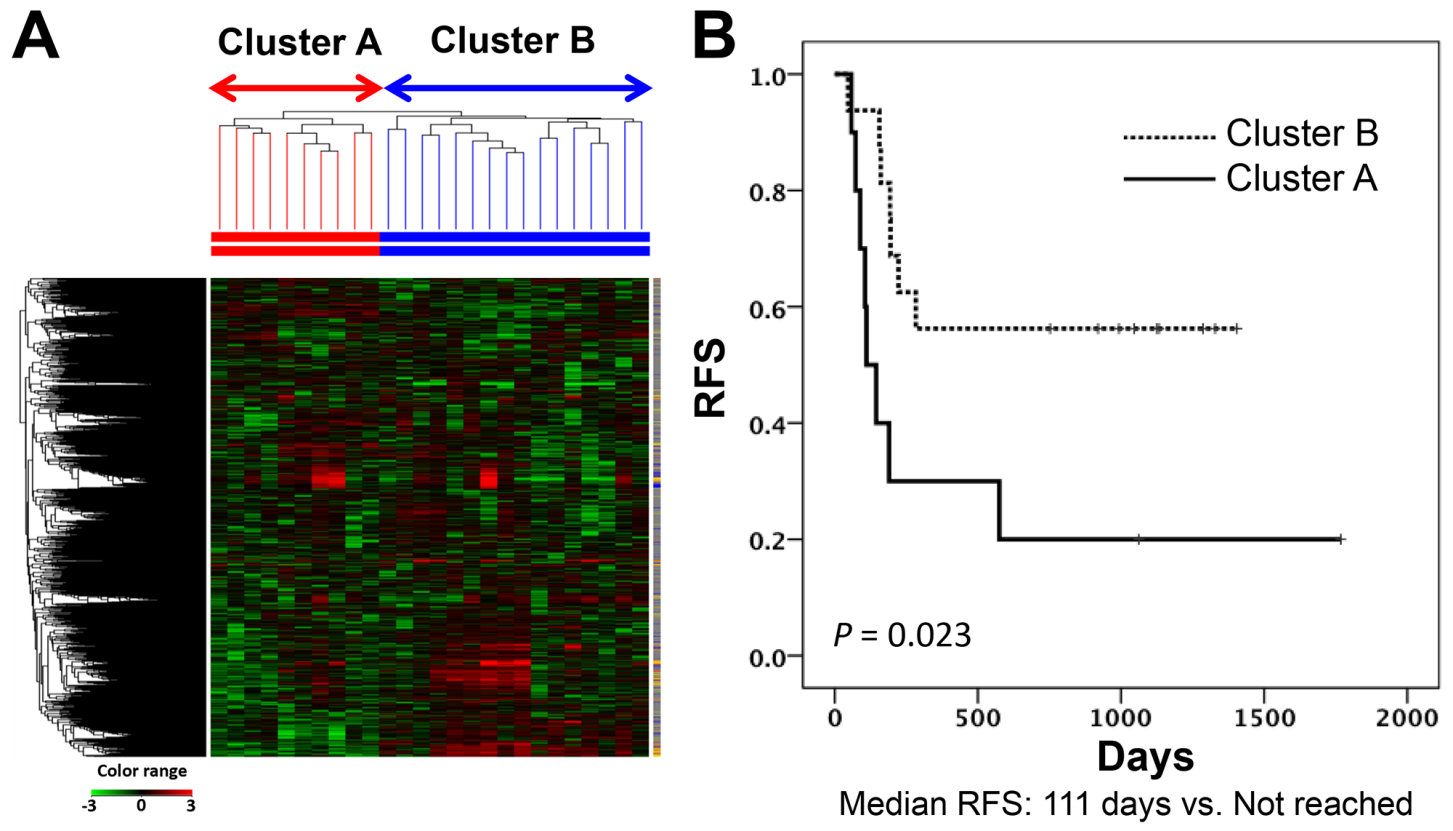
Subgrouping through unsupervised hierarchical clustering in the discovery study **(A)** Heatmap of the gene expression of 3755 probe sets used for USV clustering. **(B)** Kaplan-Meier curves for RFSs of two patient groups according to clustering analysis in the discovery cohort (Log-rank test, *P* value = 0.023). RFS, relapse-free survival.

Unsupervised hierarchical clustering for the 26 primary tumors based on cDNA microarray results formed two main clusters (cluster A and cluster B) as the first branch divisions in the dendrogram. RFS was longer in patients categorized in cluster B than in cluster A (Figure 1). Median length of follow-up was 253 days (range, 46-1768) for all enrolled patients. None of the clinicopathogical factors characterized the respective clusters (Supplementary Table 1). A total of 3755 probe sets were reliably measured by 2-fold above the global median in at least 10% of samples. Among these, 175 probe sets in cluster A and 400 in cluster B showed statistically significantly high expression based on the unpaired t-test and a greater than 2-fold change in expression against the opposite cluster. Among these probe sets, we selected the top 50 by fold change in each cluster. Comparing RFS among patient subsets dichotomized according to the median value screened out 30 prognostic genes with use of the log-rank test. Twenty-five and five genes showed high expression in cluster A and cluster B, respectively (Table 2). In addition, hierarchical clustering based on the expression of these 30 genes discriminated the patients with poorer or better prognosis (median RFS: 190 days versus not reached; log-rank test, *P* = 0.047) (Supplementary Figure 1). Thus, these findings identified a small number of genes which were associated with the prognosis of patients with TSCC.

**Table 2:** Candidate genes identified by cDNA microarray as predictive of recurrence

25 up-regulated genes in cluster A					
Probe ID	Gene symbol	Fold change	Probe ID	Gene symbol	Fold change
20205680_at	MMP10	5.12	242005_at	LOC100506377	3.24
205380_at	PDZK1	4.98	235976_at	SLITRK6	3.20
223571_at	C1QTNF6	4.75	201506_at	TGFBI	3.19
203492_s_at	FSTL3	4.09	227828_s_at	FAM176A	3.18
210248_at	WNT7A	3.79	228564_at	LOC375295	3.17
209946_at	VEGFC	3.79	212489_at	COL5A1	3.14
228762_at	LFNG	3.65	216080_s_at	FADS3	3.12
237411_at	ADAMTS6	3.47	229441_at	PRSS23	3.11
222108_at	AMIGO2	3.39	209955_s_at	FAP	3.07
233555_s_at	SULF2	3.37	243541_at	IL31RA	3.02
206230_at	LHX1	3.35	210220_at	FZD2	2.88
1559433_at	LOC149773	3.30	203726_s_at	LAMA3	2.26
203417_at	MFAP2	3.29			

5 up-regulated genes in cluster B		
Probe ID	Gene Symbol	Fold change
213240_s_at	KRT4	25.15
205623_at	ALDH3A1	24.25
210096_at	CYP4B1	16.73
1569064_at	C15orf62	10.54
229842_at	ELF3	6.94

NOTE: Clusters A and B were recognized as the favorable prognostic predicted group and the unfavorable prognostic predicted group, respectively, by unsupervised hierarchical clustering in the discovery study. Fold change was determined relative to each other group in gene expression.

### Functional enrichment analyses to explore specific annotations in cluster A and cluster B of the discovery cohort

To explore activated and inactivated pathways consisting of specific up- or down- regulated genes in each cluster of the discovery cohort, we focused on genes related to cancer-related signaling pathways. Sixty-four and 26 annotation sets were significantly enriched in clusters A and B, respectively (Supplementary Table 2). Genes specifically annotated in cluster A were related to extramatrix degradation (e.g. matrix metalloproteinases [MMPs], lymphogenesis (e.g. vascular endothelial growth factor [VEGF]) and cell-matrix interaction (e.g. integrin). In contrast, genes specifically annotated in cluster B related to tight junction interaction, such as claudin and keratin-related genes.

### Comparison of gene expression patterns between carcinoma samples in cluster A and cluster B, and normal tissue samples in the discovery cohort

**Figure 2:**
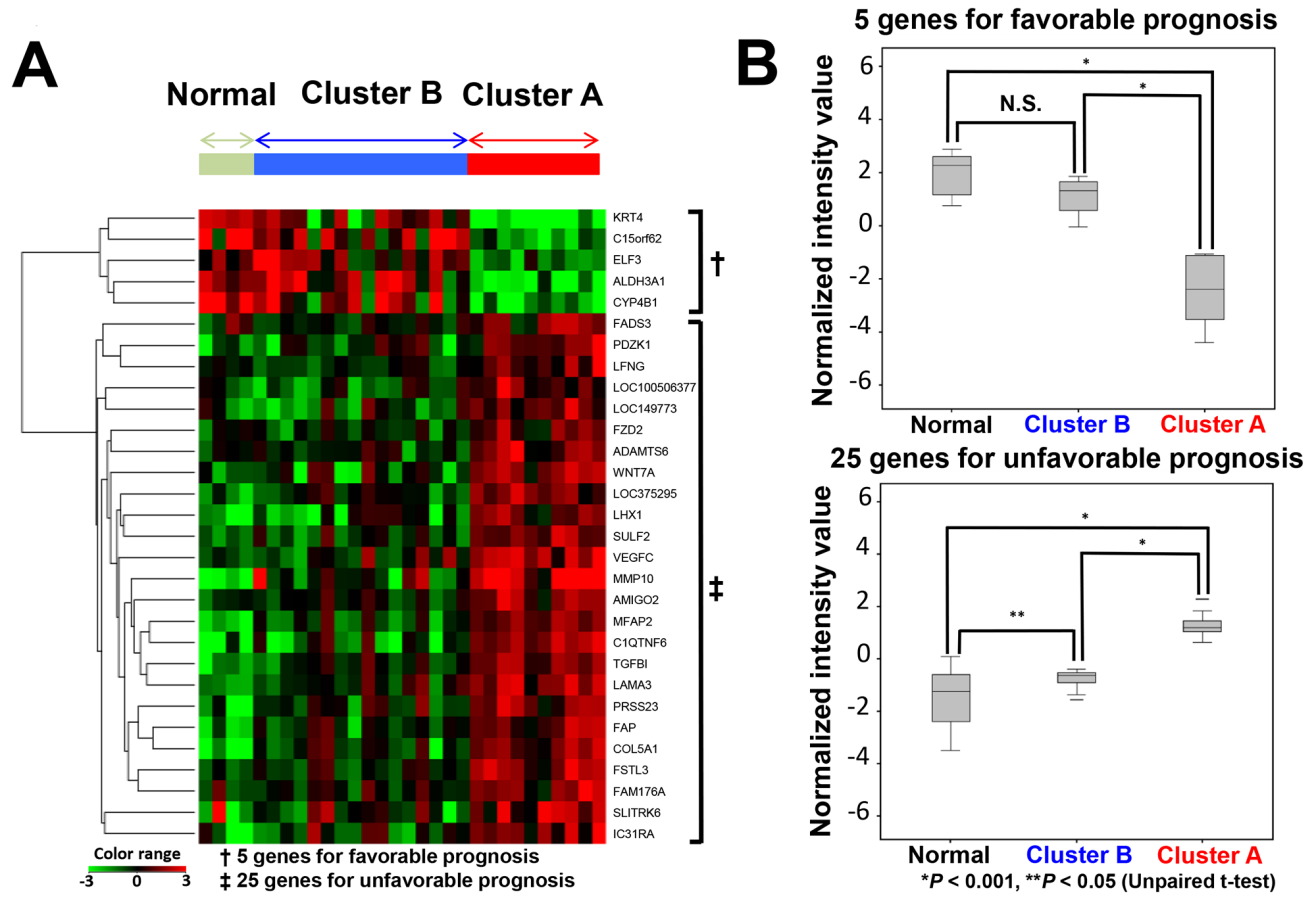
Relationship of the expression of 30 genes between cluster A, cluster B and normal tissues **(A)** Hierarchical cluster analysis of 30 samples (26 carcinoma tissues, 4 matched normal tissues) derived from 26 patients based on 30 gene expression patterns. **(B)** Normalized intensity values of predictive candidate genes of normal, cluster A and cluster B subgroups. Upper graph: five genes associated with a favorable prognosis. Lower graph: 25 genes associated with an unfavorable prognosis.

We attempted to compare expression patterns of the 30 genes between carcinoma samples of cluster A and cluster B, and in normal tissue samples in the discovery cohort. A heat map obtained from hierarchical cluster analysis showed that the gene expression pattern of cluster B was similar to that of normal tissue samples, whereas that of cluster A was quite different (Figure 2A). Additionally, the normalized intensity value of genes was examined. The aggregated normalized intensity value of five genes which indicated a favorable prognosis was comparable between cluster B and normal tissue, but significantly lower in cluster A. In contrast, the aggregated expression values of 25 genes which indicated an unfavorable prognosis was highest in cluster A, and lowest in normal tissues (Figure 2B).

### Development of Prognostic Index (PI) in the discovery cohort

We created a new PI using the 30 prognostic gene expressions and Cox regression coefficients obtained from the discovery study. Patients were classified into PI-low and PI-high groups using the median values. For RFS, the log-rank test showed a significant difference between the PI-high and PI-low groups (median RFS: 161 days versus not reached; log-rank test *P* = 0.028). Multivariate analysis was not performed because of the limited sample size (Supplementary Figure 2).

### External validation of the PI for predicting patient prognosis using three other cohorts

**Figure 3:**
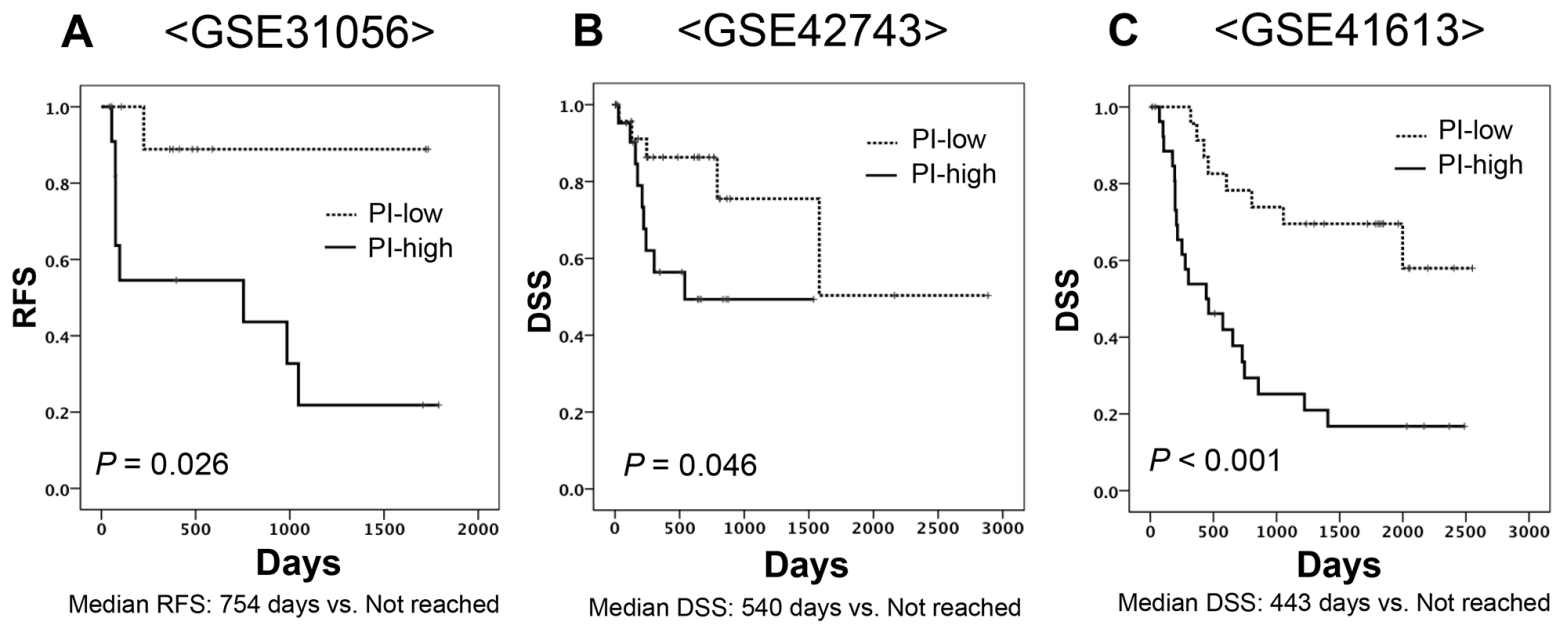
Kaplan-Meier curves of two subgroups divided based on a predictive formula in three independent cohorts **(A)** GSE31056 for RFS (Log-rank test, *P* value = 0.026), **(B)** GSE42743 for DSS (Log-rank test, *P* value = 0.046), **(C)** GSE41613 for DSS (Log-rank test, *P* value <0.001).

We applied the PI developed using the discovery cohort to the external validation cohort. Patients were classified into PI-high and PI-low groups by median value. The GSE31056 cohort showed a statistically significant gap in RFS (Figure 3A), as did the GSE42743 and GSE41746 cohorts (Figure 3B and 3C). Multivariate analysis using Cox proportional hazards to adjust for age, gender and treatment form revealed that PI was an independent prognostic marker for DSS in the GSE41613 cohort (HR 4.171, 95%CI: 1.793-9.703) (Table 3).

**Table 3:** Independent prognostic factor by multivariate analysis for DSS in GSE41613

Variable	Hazard ratio	95% CI	*P* value
**Age (years)**			
≥60	Referent	0.340-1.708	0.510
<60	0.762		
**Gender**			
Male	Referent	0.564-2.915	0.553
Female	1.282		
**Treatment**			
Mono-modality*	Referent	0.279-0.2094	0.602
Multi-modality**	0.765		
**Clustering**			
PI-low	Referent	1.793-9.703	0.001
PI-high	4.171		

*Surgery alone **Surgery with adjuvant treatment. DSS, disease specific survival; CI, confidential interval; PI, prognostic index.

### Immunohistochemical analysis as a conversion use of cDNA microarray data for the selection of TSCC patients with better prognosis

The *KRT4* gene was selected as a candidate for identifying TSCC patients with a better prognosis by cDNA microarray. Cytokeratin 4 (CK4) encoded by the *KRT4* (keratin 4) gene was elucidated by immunohistochemical staining in a separate cohort of 127 TSCC patients to evaluate the conversion use of cDNA microarray data. The cut-off value for CK4 protein positivity rate was determined using receiver operating characteristic (ROC) analysis of the relationship between CK protein positivity and *KRT4* gene expression in the discovery data, and found to be 5% (Supplementary Figure 3). The sensitivity and specificity of CK4 protein positivity by immunohistochemical staining for predicting high *KRT4* gene expression and low PI were 46% and 92%, and 38% and 85%, respectively. In addition, positive and negative predictive values in the discovery cohort data were 86% and 63%, and 71% and 58%, respectively.

**Figure 4:**
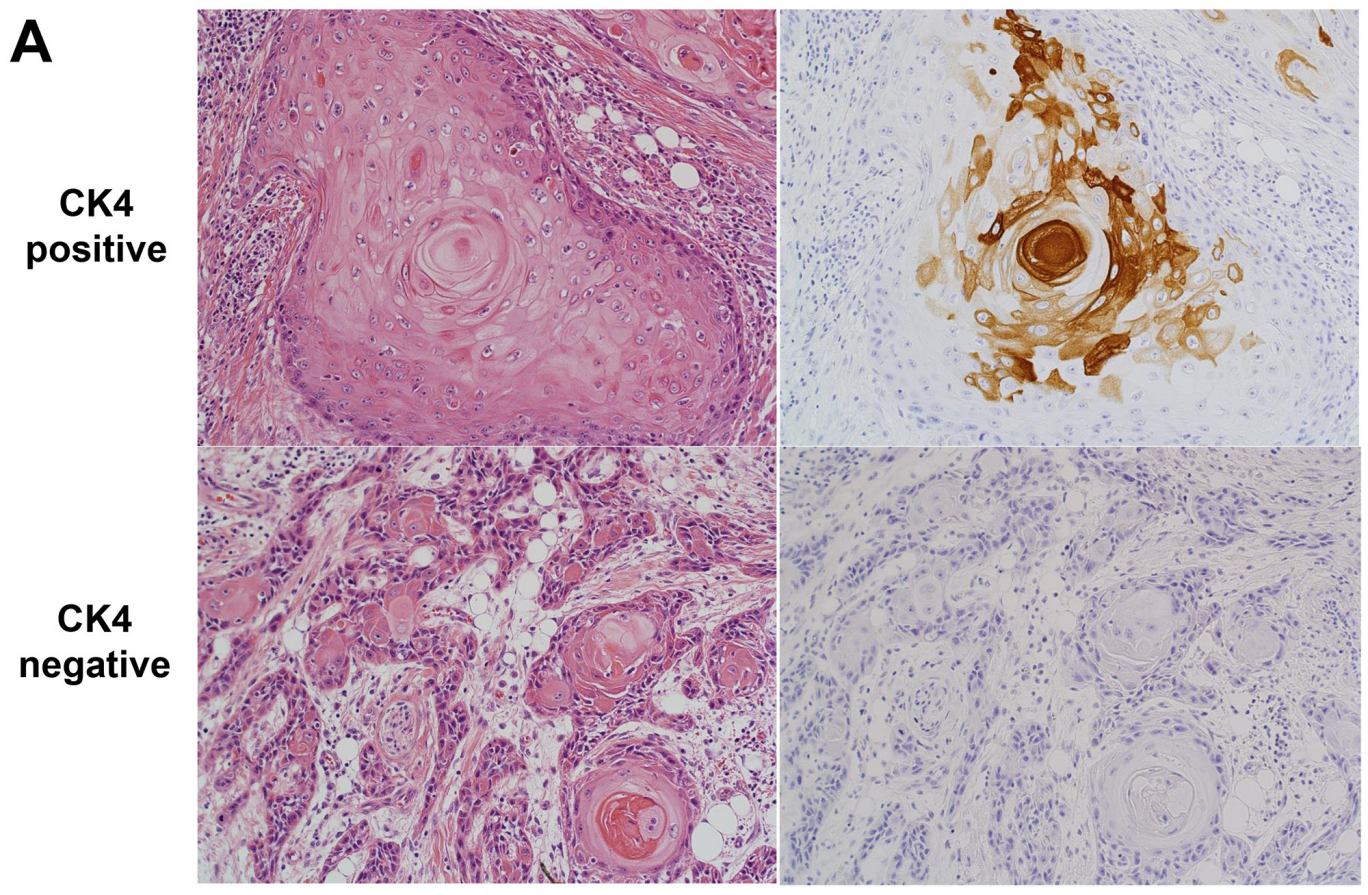

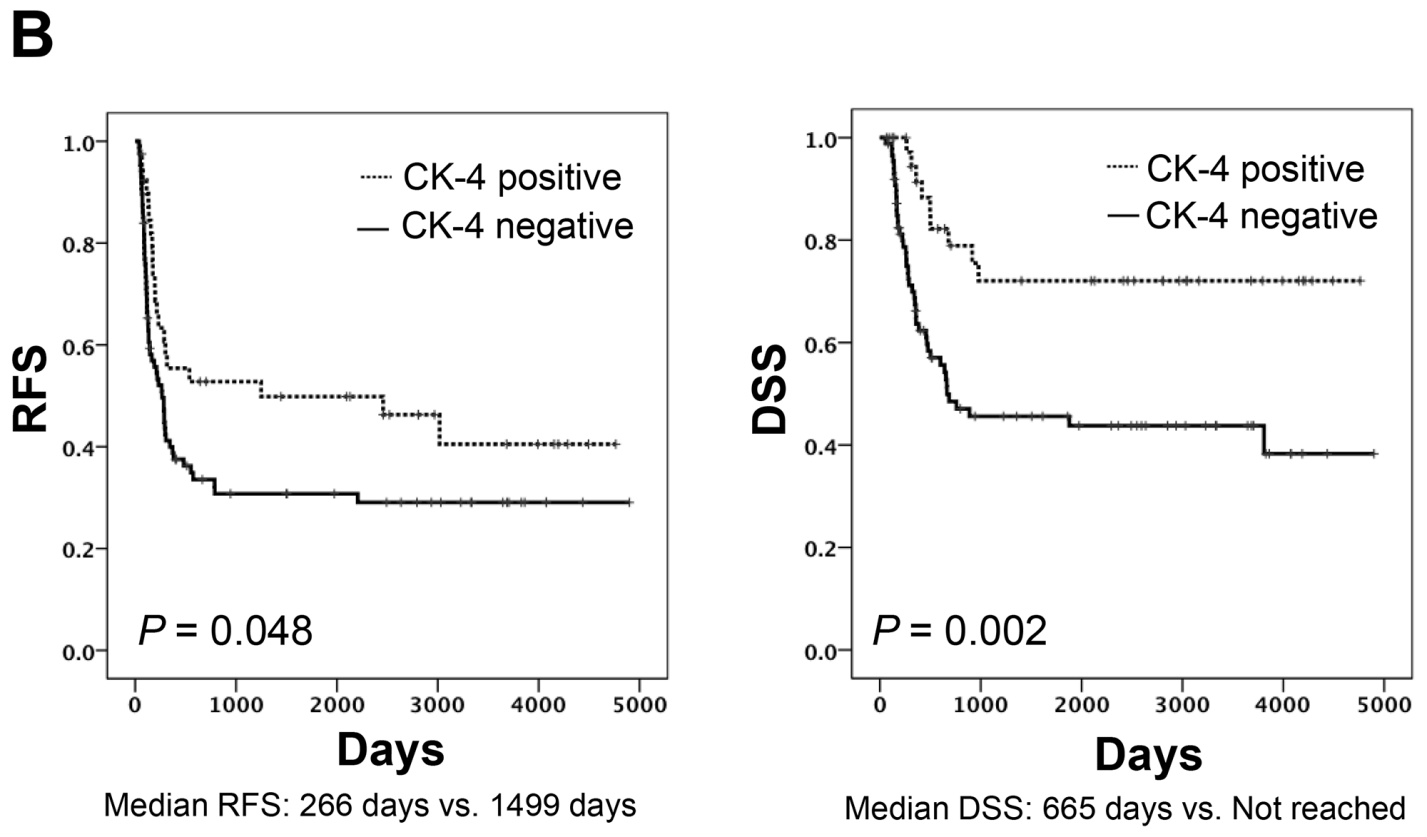
Relationship between CK4 protein expression, RFS and DSS **(A)** Micrograph of a representative TSCC expressing CK4 protein detected byimmunohistochemical staining. Left, hematoxylin & eosin; right, CK4. **(B)** Kaplan-Meier curves drawn according to CK4 protein expression by immunohistochemical staining. Left, RFS, Log-rank test, *P* value = 0.048. Right, DDS Log-rank test, *P* value = 0.002.

Forty patients (31%) were judged to be CK4-positive. Significant differences in RFS and DSS were seen by CK4 status (Figure 4A and 4B). Multivariate Cox regression analysis in 127 TSCC patients revealed that CK4 positivity was an independent favorable prognostic factor in TSCC patients for RFS (HR 0.591, 95% CI: 0.354-0.988) and DSS (HR 0.333, 95%CI: 0.159-0.697) (Table 4). Among another 80 patients who developed recurrence, those who were positive for CK4 had a significantly longer DSS after recurrence than those who were CK4-negative (Log-rank test, *P* = 0.036) (Supplementary Figure 4). In addition, multivariate analysis identified CK4-positivity as an independent predictor for disease-specific death from recurrence (HR 0.418, 95%CI: 0.201-0.870), as well as factors including salvage surgery and systemic chemotherapy administration (Supplementary Table 3). Significant correlations between CK4 protein expression and several clinicopathological factors of patients were seen in the validation cohort. CK4-positivity was significantly correlated with histological differentiation (*P* = 0.029), while CK4-negativity was significantly correlated with vascular invasion (*P* = 0.030) (Supplementary Table 4). The Kaplan–Meier curves for differentiation revealed no significant difference between well-differentiated and other types in RFS (log-rank *P*=0.245) or DSS (log-rank *P*=0.124) (Supplementary Figure 5).

**Table 4:** Association between potential prognostic factors, and RFS and DSS in the validation study of immunohistochemical staining

Variable	n	RFS			DSS		
		Hazed ratio	95%CI	*P*-value	Hazard ratio	95%CI	*P*-value
**Age (years)**							
≥45	101	Reference	0.687-2.111	0.515	Reference	0.279-1.441	0.276
<45	26	1.205			0.634		
**Gender**							
Male	97	Reference	0.449-1.347	0.369	Reference	0.545-2.020	0.886
Female	30	0.777			1.049		
**Treatment**							
Mono-modality*	112	Reference	0.276-1.193	0.137	Reference	0.257-1.506	0.293
Multi-modality**	15	0.574			0.623		
**pTstage**							
T1-2	36	Reference	0.655-1.758	0.780	Reference	0.657-2.160	0.564
T3-4	91	1.073			1.191		
**pNstage**							
N0-1	45	Reference	1.053-2.970	0.031	Reference	1.064-3.885	0.032
N2-3	82	1.769			2.033		
**Positive Margin**							
Absent	107	Reference	0.826-2.702	0.184	Reference	0.848-3.433	0.134
Present	20	1.494			1.707		
**Extranodal spread of lymph node metastasis**							
Absent	96	Reference	0.919-2.506	0.103	Reference	0.936-3.266	0.08
Present	31	1.518			1.748		
**CK4**							
Negative	87	Reference	0.354-0.988	0.045	Reference	0.159-0.697	0.004
Positive	40	0.591			0.333		

NOTE: Analysis uses a multivariate cox regression models for RFS and DSS using variables risk factors including CK4 protein expression. *Surgery alone **Surgery with adjuvant treatment. RFS, relapse-free survival; DSS, disease specific survival; CI, confidential interval; CK4, Cytokeratin 4.

### Real-time quantitative polymerase chain reaction (RT-qPCR) of *KRT4* gene in the discovery cohort

Cases with higher expression of the *KRT4* gene from DNA microarray data also showed higher expression of *KRT4* gene by RT-qPCR. Further, cases with lower microarray expression of the *KRT4* gene showed lower expression of *KRT4* gene by RT-qPCR. Accordingly, there was a statistically significant relationship between the two groups in the value of *KRT4* expression (*P* =0.0128) (Supplementary Figure 6).

## DISCUSSION

This DNA microarray study demonstrated that TSCC patients could be differentiated into two groups with different prognoses by two molecular subtypes, regardless of clinicopathological characteristics. We then used this finding to identify 30 prognostic genes associated with recurrence and created a formula to predict prognosis in individual patients. In a validation study, clustering based on this formula discriminated two subgroups with a significantly different RFS. The multivariable gene and protein expression models established in this study may help identify patients requiring more intensive treatment and improve outcomes of TSCC patients.

The DNA microarray in this study demonstrated that TSCCs in cluster A behaved as highly malignant, with stubborn recurrence and frequent metastasis. Of note, several differentially expressed genes in cluster A, including MMP-10 and VEGFC, are responsible for degradation of extracellular matrix (MMP-10) and lymphogenesis and angiogenesis (VEGFC) [12–14]. Interestingly, the effectiveness of inhibitors targeting the protein encoded by these genes on TSCC cells has already been demonstrated in both *in vitro* and *in vivo* assays [12, 15, 16], although further studies with more specific inhibitors are required before these can be implemented into clinical care [17]. In contrast, the TSCCs in cluster B were characterized as having similar gene expression patterns to normal squamous epithelium. Claudins, a family of molecules with relatively higher levels in cluster B than A, are major structures in the tight junctions in human epithelia [18]. It has been shown that epithelial–mesenchymal transition (EMT)-related transcriptional factor, which is induced by Transforming Growth Factor-β (TGF-β), down-regulates the expression of claudins [19]. Recent studies have linked claudins to disease aggressiveness, poor prognosis and EMT in several tumors [20–23]. Lohavanichbutr et. al developed a gene expression signature consisting of 13 genes to improve risk assessment in early- and advanced-stage human papolloma virus (HPV)-negative oral squamous cell carcinoma (OSCC) patients [24]. The genes identified in their study were not consistent with those in our present study, presumably because of the use of different methods in statistical analysis and clinical staging. However, many of the genes in their study have also been implicated in cell invasion, motility, focal adhesion and proliferation. In Lohavanichbutr’s paper, the HR for OSCC-specific mortality was 3.12 (95% CI, 1.24–7.87) after adjustment for age, gender, and tumor stage (pStage I-IV). In contrast, our study included only advanced TSCC patients (pStage III/IV). Nevertheless, HR remained significant after adjustment for age, gender, and adjuvant therapy status at 4.171 (95% CI, 1.793–9.703) for TSCC-specific mortality, which was equal to or higher than that of Lohavanichbutr. Furthermore, our multivariate analysis included adjuvant therapy status as a potential confounding factor. In summary, our model showed that a high PI was a novel and high risk factor for cancer-related death in surgically treated advanced TSCC patients, regardless of the presence or absence of current adjuvant therapy. TSCC patients with a high PI might require more aggressive adjuvant therapy; nevertheless, use of our PI to establish a novel therapeutic strategy would require a prospective study.

*KRT4*, which encodes CK4 protein, was also unregulated in cluster B, and is known to be down-regulated in OSCC and esophageal squamous cell carcinoma compared to normal squamous epithelium [25, 26]. Tieneke et al reported that low expression of CK4 in the surgical margins showed a highly significant association with the development of local recurrence [27]. Mutation of the *KRT4* gene leads to the development of white sponge nevus, which is characterized by oral leukoplakia [28]. Thus, although the direct relationship between its expression and tumor characteristics remains unclear, CK4 protein is reported to be expressed in differentiated and keratinized squamous cell carcinoma cells. However, our results suggest that some upstream signals associated with TSCC aggressiveness might be reflected in the down-regulation of CK4 protein.

The identification of biomarkers through translational research would have little value unless the results could be used in routine clinical decision-making. Genomic tests, including microarray, are expensive and limited on by the kind of sample that can be tested. The development of easier methods which take account of tumor heterogeneity and cost-effectiveness will therefore be of global benefit. This in turn warrants the conversion of gene expression data to information on protein expression status. One example of this is the Mammostrat test, an immunohistochemical multigene assay, which enables the semi-quantitative scoring of five genes to stratify the risk of recurrence of breast cancer [29–31]. In our study, *KRT4* gene expression was alternatively reflected in immunohistochemical staining, and considered useful in predicting a better prognosis in the validation cohort. This suggests that this costly and limited genetic analysis can be converted to a routine pathological examination.

Given that our study was conducted under a retrospective design, additional prospective studies with eligibility criteria applicable to clinical trials are needed to confirm that the gene signatures based on our results accurately predict outcomes in TSCC patients. We also need to determine whether an intensification of postoperative treatment for patients with molecularly unfavorable tumors will significantly improve rates of relapse and death, and whether these candidate biomarkers include novel drug targeting candidates. If they do include novel candidates, detailed analysis will likely support the development of personalized and customized therapy for TSCC. This study also produced a number of other interesting findings. Several reports have described the influence of a young age at the time of TSCC diagnosis on prognosis [32, 33]. In our study, young age was not one of the worse prognostic factors. Our immunohistochemical analysis did not identify all of the biological factors associated with prognosis in young patients. However, our cohort was highly selected and homogenous, and although young age might not be among the worst prognostic factors for TSCC, further analysis is required.

In this study, we used a comprehensive discovery and validation process in separate samples to identify a biomarker which distinguished TSCC patients with poor prognosis. In addition, we successfully translated data for gene expression profiling to protein expression profiling using immunohistochemical staining. The biomarker identified in this study is suitable for use in routine examination, suggesting that our study may open the door to the development of more personalized treatment and better patient prognosis in patients with TSCC.

## MATERIALS AND METHODS

This study was conducted using discovery and validation study cohorts. In the discovery study, we first conducted gene expression profiling of clinico-pathologically homogeneous advanced TSCC tissue samples using cDNA microarrays. In the validation study, we then examined whether the prognostic index constructed from genes identified in the discovery study could be validated using microarray data sets from three other publicly available cohorts. In addition, we examined whether the gene expression data obtained by cDNA microarray could be converted to immunohistochemical data of protein expression using other TSCC patients, in light of the cost and sample quality limitations involved in applying the signature to clinical practice.

### Patients and data sets

The discovery cohort for cDNA microarray consisted of patients with loco-regionally advanced TSCC who had undergone surgical resection of the primary tumor at the National Cancer Center Hospital East (NCCHE) between November 2009 and January 2013 and were pathologically diagnosed as pStage III/IV. Surplus primary tumor and non-tumor tissue specimens were immediately retrieved after surgical resection, and registered and stocked in the tissue bank of NCCHE. A total of 134 tumor samples from 134 patients were collected in the head and neck cancer section during this period, of which 40 were from 40 patients with TSCC. Among these, we excluded samples from a recurrent lesion (n=1) and from patients diagnosed with pStage I/II disease (n=6) or with other synchronous cancers (n=1). From the remaining 32 patients, 26 were randomly selected by a certificated pathologist (S.F.) who was blinded to patient clinical outcome as the discovery cohort. Meanwhile, we also selected published microarray gene expression profile data and accompanying prognostic data from three different OSCC patient cohorts from the Gene Expression Omnibus (http://www.ncbi.nlm.nih.gov/geo/; GEO-NCBI) for the validation study. These gene expression data were obtained using the same microarray platform as that used in the discovery study. We then selected data from tumor samples based on the following criteria, if applicable: (1) tumor stage was stage III/IV (except for the GSE31056 cohort due to the limited number of patients); and (2) treatment consisted of surgery with or without postoperative therapy. This produced data for 123 tumor samples for the first validation study (Supplementary Table 5) (as described elsewhere in http://www.ncbi.nlm.nih.gov/geo/; accession number GSE31056, GSE42743 and GSE41613) [24, 34].

In addition, the supplementary immunohistochemical analysis study was performed using an additional 127 patients who were compatible with the discovery cohort in clinicopathological factors and had been treated between January 2002 and March 2009 at NCCHE.

Histological classification and TNM staging of the tumor specimens were performed according to the WHO classification and Union for International Cancer Control (UICC), respectively [35, 36]. RFS was defined as the period from the date of surgery to relapse or death, whichever came first. Meanwhile, DSS was defined as the period from the date of surgery to cancer-specific death. Data regarding clinicopathological and prognostic data of enrolled patients were gathered from medical records.

This study was approved by the Institutional Review Board of the National Cancer Center Institute on June 4, 2012 (Registration No. 2012-013) and was conducted in accordance with the guidelines of the Helsinki Declaration.

### Gene expression profiling analysis using cDNA microarray

RNA was extracted from surgically resected tissues using an RNA isolation kit (Rneasy Plus Mini Kit/EZ1 RNA Universal Tissue Kit, Qiagen). We used commercially available and widely accepted GeneChip Human Genome U133 Plus 2.0 arrays (Affymetrix) using GCS 3000Dx, as supplied through the official distributor Sysmex Corporation (Kobe, Japan). The primary expression microarray data are available at GEO under the accession number GSE 78060. Gene expression data were analyzed with GeneSpring GX12.6 (Agilent Technologies). Raw data were summarized using MAS5 and converted to log2 values. The values were then standardized by the respective normalized intensity value as calculated by dividing by the median of all genes’ intensity values to permit gene selection and intercomparison of gene expression levels. For unsupervised hierarchical clustering, we used probe sets that were reliably measured and varied by 2-fold above the global median in at least 10% of samples, followed by average linkage clustering with Pearson correlation distance. We did not use any clinical or pathological parameters for this clustering, and the two groups are accordingly discriminated solely based on the profiling of differentially expressed genes (DEGs). To highlight the distinct functional features of the subgroups with different prognoses, gene set enrichment analysis (GSEA) using MSigDB v5.0 was also applied [37, 38]. GSEA using C2 (curated gene sets) consisting of 4725 gene sets was carried out to estimate the functional annotation enriched in each subgroup. Gene sets showing significant enrichment in each subgroup of the discovery cohort were selected based on an adjusted *P*-value < .05 and a false discovery rate (FDR) < .05 with Benjamini-Hochberg multiple correction.

### Immunohistochemical analysis

To confirm the results obtained by cDNA microarray using protein expression levels, expressions of several proteins encoded by differentially expressed candidate prognostic genes were elucidated by immunohistochemical staining in a different cohort. Finally, CK4 protein was selected for further immunohistochemical analysis, after trials to investigate the specificity and sensitivity of remaining antibodies. For each case, sections were cut at a thickness of 4 μm from formalin-fixed and paraffin-embedded (FFPE) tissue blocks, including tumor-representative areas. After endogenous peroxidase activity was blocked with 0.3% H_2_O_2_ in methanol, antigen retrieval was performed by immersion in buffer (S2367, Target Retrieval Solution, pH 9; Dako) in a microwave oven for 10 minutes at 95°C. The anti-CK4 antibody (ab9004; Abcom) was diluted at 1:500 in antibody diluent (S3022, Antibody Dilution with Background Reducing Components; Dako) and the sections were incubated with the antibody overnight at 4°C. The slides were then reacted with a peroxidase-labeled secondary antibody (K4000, EnVision/HRP system; Dako) and visualized as a brown reaction product by applying 3,3’-diaminobenzidine (DAB) solution (K3467, DAB_+_ Liquid; Dako). Immunohistochemical staining for light microscopy was performed by a certificated pathologist (S.F.) who was blinded to patient clinical outcome, and the positive percentage of carcinoma cells for each case was elucidated.

### Real-time quantitative polymerase chain reaction (RT-qPCR)

To confirm the expression of *KRT4*, we examined *KRT4* gene expression by RT-qPCR and compared it with the data obtained from DNA microarray analysis in the discovery cohort. Nine samples were available for RT-qPCR. Glyceraldehyde-3-phosphate dehydrogenase (GAPDH) was used as an endogenous control to normalize each sample. The primers for *KRT4* and *GAPDH* are listed in Supplementary Table 6.

### Statistical analysis

In the discovery study, unsupervised clustering analysis identified two principal clusters. Differentially expressed probe sets which distinguished the two clusters were selected based on the following criteria: (1) differentially expressed probe sets that had a gene symbol-level annotation versus the other group at *P* < .05 (calculated using unpaired t-test with Benjamini and Hochberg multiple correction) and a >2-fold change in expression; (2) the top 50 probe sets by fold change in each cluster; and (3) gene expression level was dichotomized according to the median value, with a *P*-value on comparison of RFS using the log-rank test of less than 0.1. PI was defined as the linear combination of gene expression levels (log10 scale) and their regression coefficient estimated from the univariate Cox model (log10 scale) (Supplementary Figure 7). By the end of this step, we extracted the prognostic genes and weighted each gene according to its prognostic value in improving outcome prediction accuracy. Accordingly, PI enables the risk of recurrence and cancer-specific death to be determined for individual patients. Theoretically, prognosis should worsen as PI increases. In the validation study, we used the PI constructed in the discovery study to dichotomize the patients into two groups, then compared RFS and DSS curves for the two subgroups using the log-rank test and multivariate Cox regression analyses.

As a supplementary analysis, we performed Cox regression analyses to evaluate if the groups dichotomized by certain gene expression levels identified in the discovery and validation cohorts could be substituted with the dichotomous group identified by the immunohistochemical staining of CK4. The predictability of *KRT4* gene expression and PI were evaluated by calculating the sensitivity, specificity, and positive and negative predictive values of CK4 protein positivity by immunohistochemical staining. The multivariate analysis included the possible confounding variables of age at treatment; gender; treatment mode (surgery alone versus surgery with adjuvant treatment); pathological T stage; pathological N stage; surgical margin status; and the presence or absence of extranodal spread of lymph node metastasis, lymphovascular and perineural invasion. Clinicopathological variables were compared by CK4 status using the paired t-test. Survival curves were estimated using the Kaplan-Meier method and compared using the log-rank test. All statistical analyses except gene expression profiling analyses were carried out using SPSS software, version 21 (IBM Corporation, NY), and all *P* values were two-sided.

## SUPPLEMENTARY MATERIALS FIGURES AND TABLES







## Abbreviations

TSCC: tongue squamous cell carcinoma
RFS: relapse-free survival
DSS: disease-specific survival
MMP: matrix metalloproteinase
VEGF: vascular endothelial growth factor
PI: prognostic index
GSE: gene expression omnibus series
CK4: cytokeratin 4
KRT4: keratin 4
ROC: Receiver operating characteristic
RT-qPCR: Real-time quantitative polymerase chain reaction
EMT: epithelial–mesenchymal transition
TGF-β: Transforming Growth Factor-β
HPV: human papolloma virus
OSCC: oral squamous cell carcinoma
HR: hazard ratio
NCCHE: National Cancer Center Hospital East
GEO: gene expression omnibus
UICC: Union for International Cancer Control
DEGs: differentially expressed genes
GSEA: gene set enrichment analysis
FDR: false discovery rate
FFPE: formalin-fixed and paraffin-embedded
DAB: diaminobenzidine
GAPDH: Glyceraldehyde-3-phosphate dehydrogenase
