# Supplementary material for: Gene expression profiling to predict recurrence of advanced squamous cell carcinoma of the tongue: discovery and external validation
Source: Oncotarget. 2017 Jun 27;8(37):61786–99. doi: 10.18632/oncotarget.18692 (PMC5617464; doi:10.18632/oncotarget.18692)
Supplement: Supplementary file 1 [file oncotarget-08-61786-s001.pdf]

Gene expression profiling to predict recurrence of advanced squamous cell carcinoma of the tongue: discovery and external validation

SUPPLEMENTARY MATERIALS

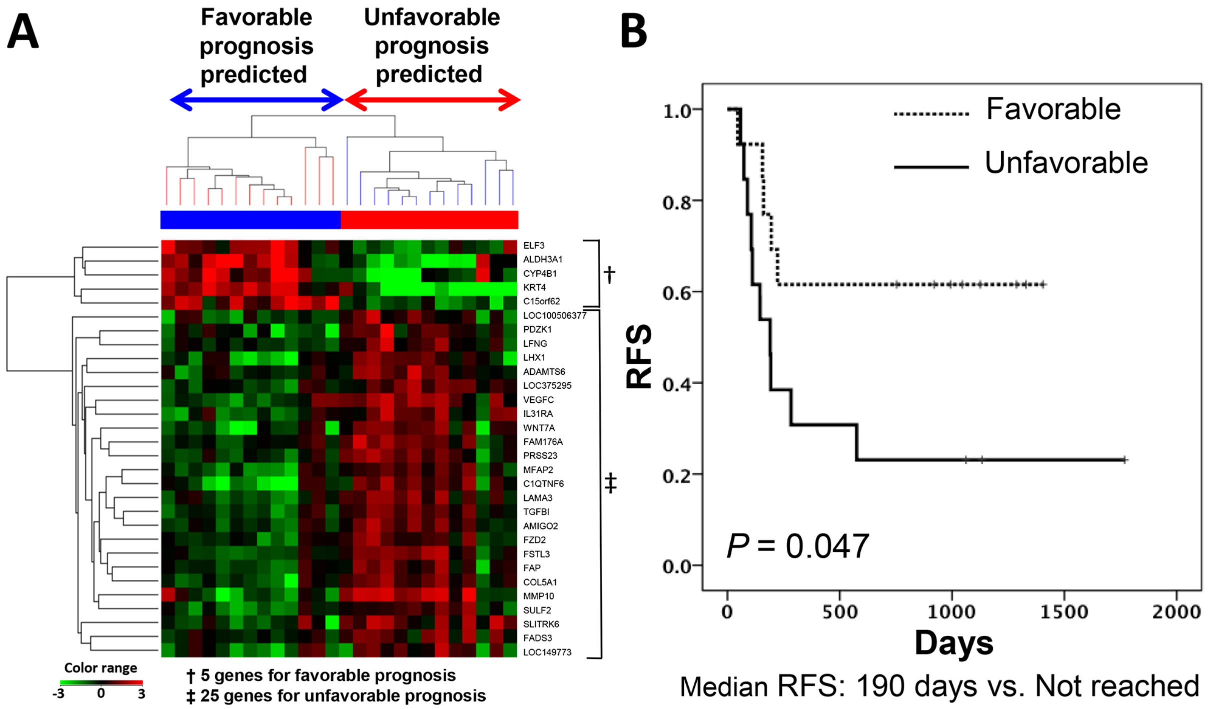

Supplementary Figure 1: Subgrouping through hierarchical clustering based on expression of 30 predictive genes in the discovery study.

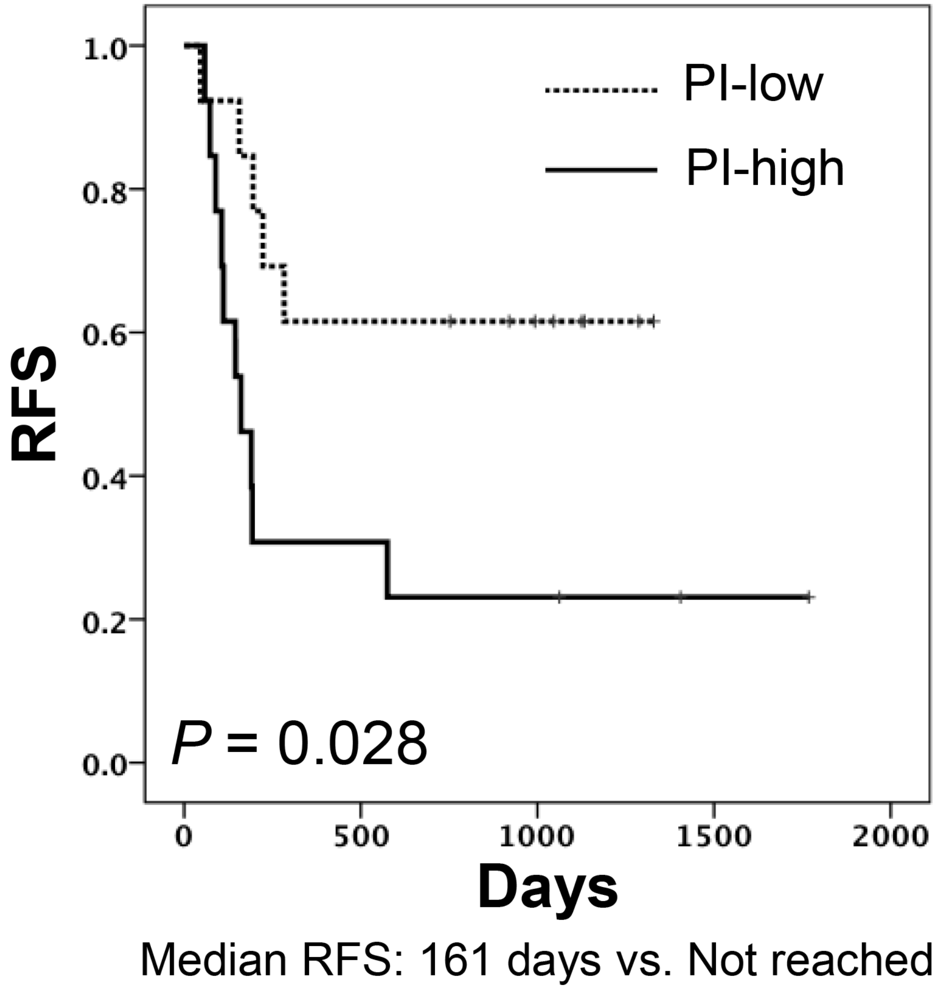

Supplementary Figure 2: Kaplan-Meier curves of the two subgroups divided based on predictive formula in the discovery study.

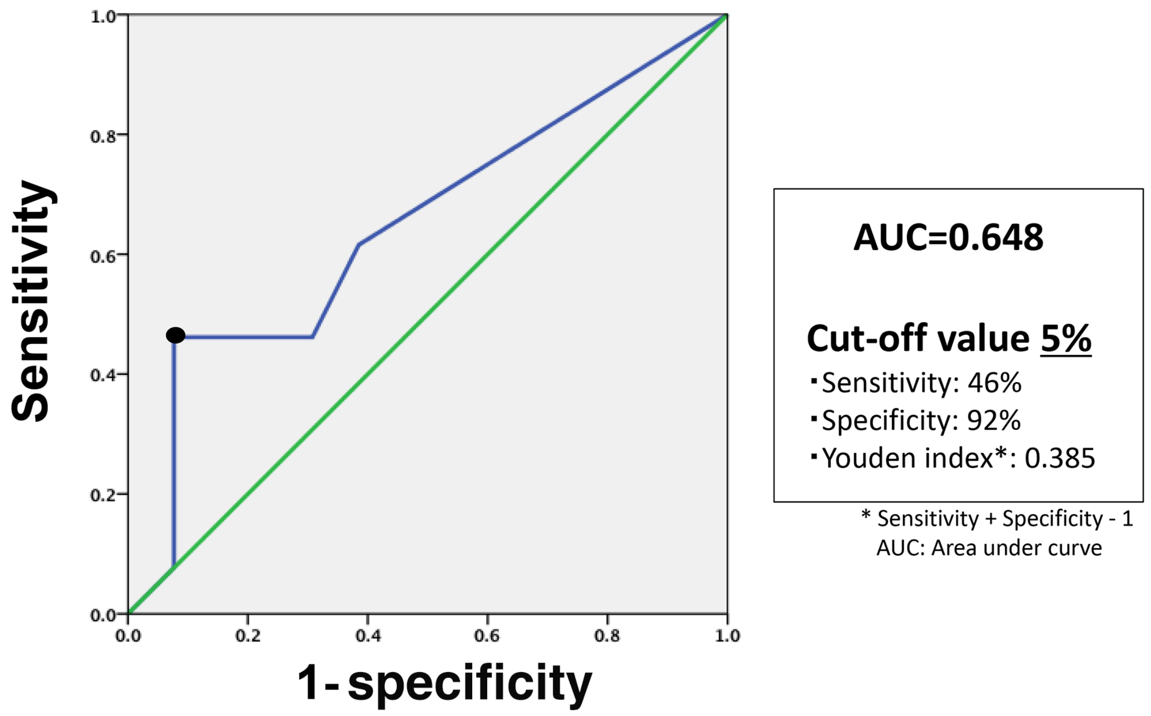

Supplementary Figure 3: Receiver operating characteristic (ROC) analysis for relationship between CK-4 protein positivity and KRT4 gene expression in the discovery study.

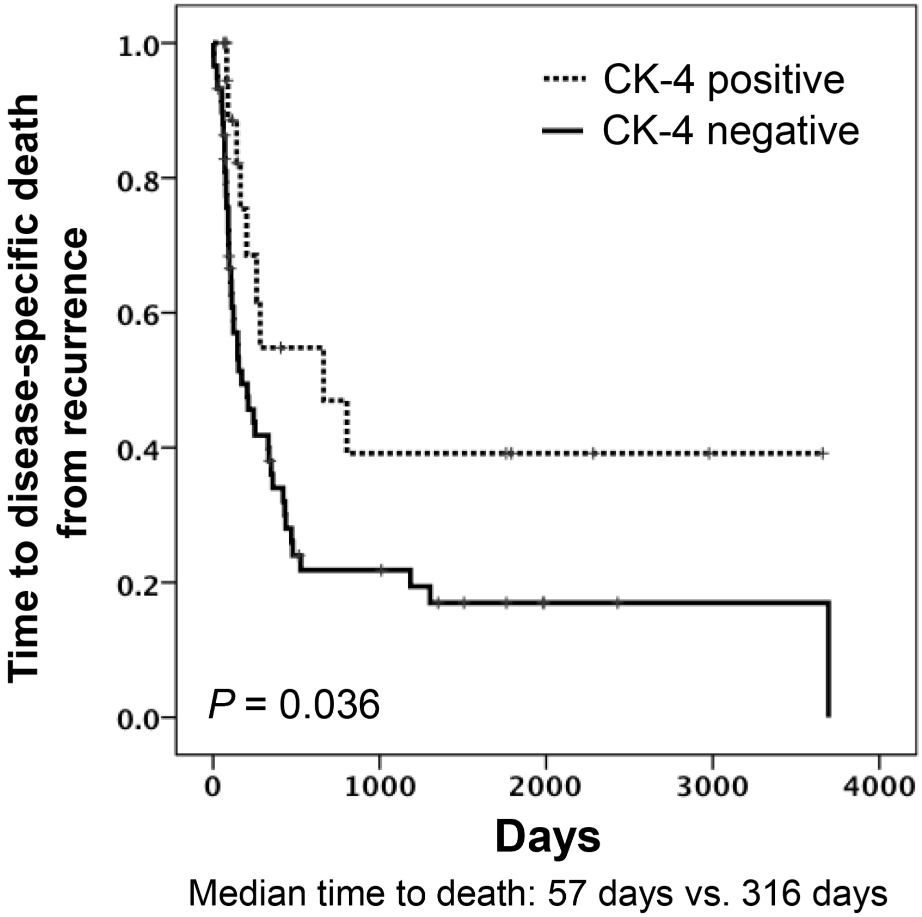

Supplementary Figure 4: Kaplan-Meier curves for the length of time after recurrence until cancer-specific death according to CK-4 protein expression by immunohistochemical staining.

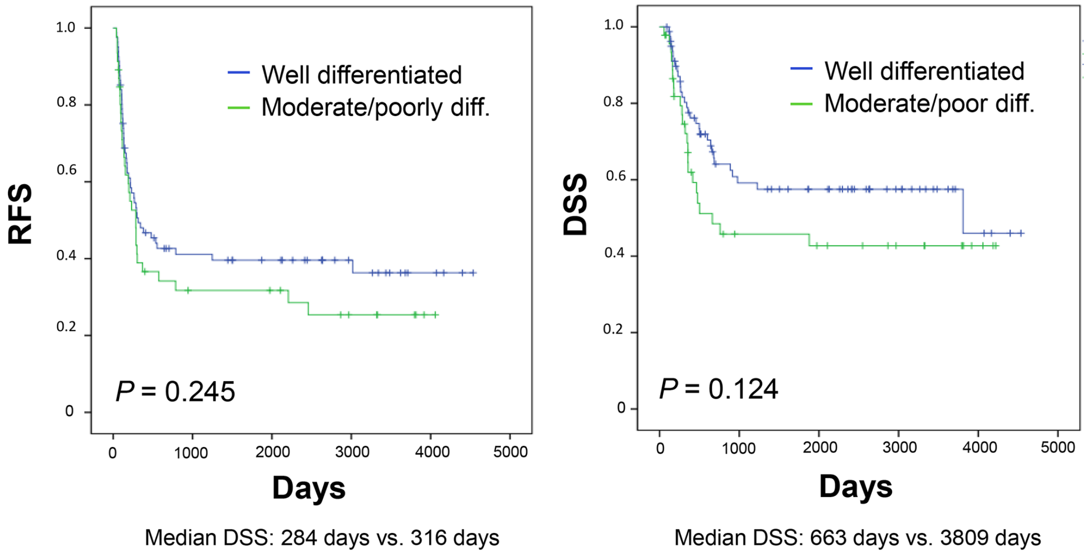

Supplementary Figure 5: Kaplan-Meier curves according to tumor differentiation in the validation study of immunohistochemical staining.

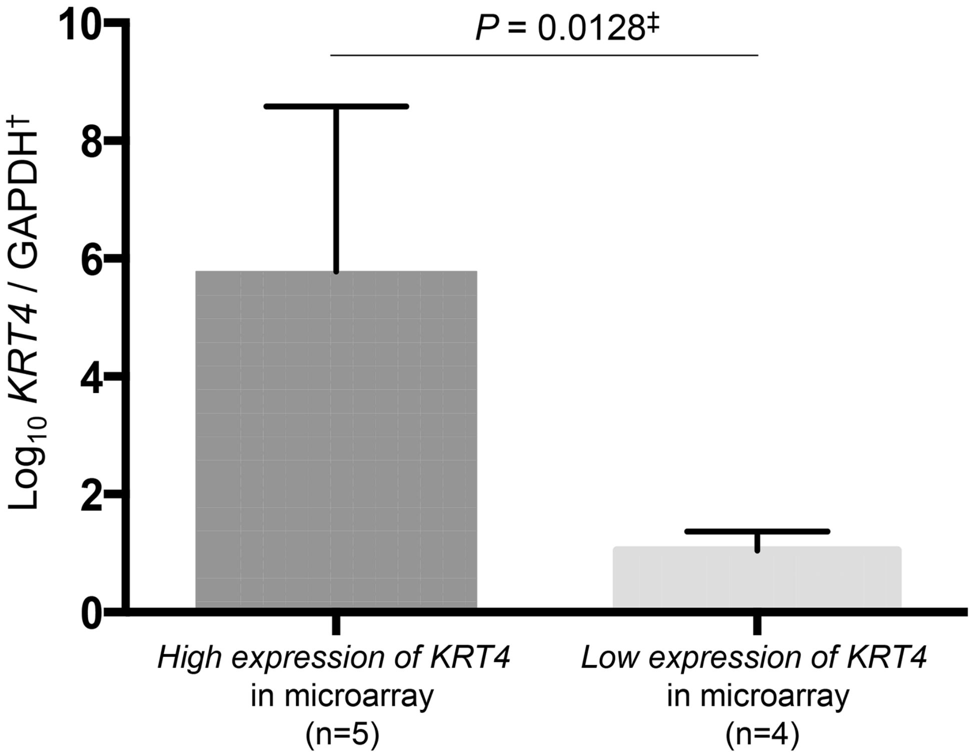

†*KRT4* value was divided by the GAPDH value ‡Unpaired t-test

Supplementary Figure 6: Relationship of the expression of 30 genes between cluster A, cluster B and normal tissues.

$$\text{Prognostic index (PI)} = \sum_{i=1}^{30} W_i X_i$$

Where
W_i is the regression coefficient estimated from the univariate Cox model in log10 scale
X_i is the raw gene expression value in log10 scale

25 up-regulated genes in cluster A
(Unfavorable group)

Probe ID	Gene symbol	Regression coefficient	Probe ID	Gene symbol	Regression coefficient
229441_at	PRSS23	7.128	209955_s_at	FAP	3.015
205680_at	MMP10	5.759	205380_at	PDZK1	2.991
203492_s_at	FSTL3	4.933	1559433_at	LOC149773	2.978
237411_at	ADAMTS6	4.890	216080_s_at	FADS3	2.918
212489_at	COL5A1	4.472	206230_at	LHX1	2.859
203726_s_at	LAMA3	3.526	210248_at	WNT7A	2.847
228762_at	LFNG	3.459	227828_s_at	FAM176A	2.847
210220_at	FXD2	3.346	209946_at	VEGFC	2.801
223571_at	C1QTNF6	3.320	242005_at	LOC100506377	2.579
233555_s_at	SULF2	3.179	243541_at	IL31RA	2.512
222108_at	AMIGO2	3.125	228564_at	LOC375295	2.477
203417_at	MFAP2	3.125	235976_at	SLITRK6	2.458
201506_at	TGFBI	3.125			

5 up-regulated genes in cluster B
(Favorable group)

Probe ID	Gene Symbol	Regression coefficient
205623_at	ALDH3A1	0.310
229842_at	ELF3	0.334
213240_s_at	KRT4	0.344
210096_at	CYP4B1	0.370
1569064_at	C15orf62	0.413

Supplementary Figure 7: Prognostic index.

Supplementary Table 1: Clinicopathologic characteristics of patients in the discovery study classified according to unsupervised hierarchical clustering.

See Supplementary File 1

Supplementary Table 2: Functional Enrichment Analyses in the discovery cohort.

See Supplementary File 2

Supplementary Table 3: Association between potential prognostic factors and time to disease-specific death from recurrence in the validation study of immunohistochemical staining

Variable	n	Time to disease-specific death from recurrence		
		Hazard ratio	95% CI	*P* value
**Age at recurrence**†		0.994	0.974-1.014	0.559
**Gender**				
Male	62	Reference	0.669-2.764	0.395
Female	18	1.360		
**Recurrence location**				
Loco-regional	54	Reference	0.507-1.823	0.904
Distant metastasis	26	0.961		
**Salvage surgery**				
No	58	Reference	0.049-0.237	<0.001
Yes	22	0.108		
**Systemic chemotherapy**				
No	64	Reference	0.100-0.462	<0.001
Yes	16	0.215		
**CK4**				
Negative	59	Reference	0.201-0.870	0.02
Positive	21	0.418		

†Continuous variable. CI, confidence interval; CK4, cytokeratin4.

Supplementary Table 4: Correlation of CK4 expression with clinicopathological features

	CK4-positive (n=40)	CK4-negative (n=87)	*P* value
**Age† Mean ±SD (range)**	54.13 ±15.8 (24-77)	58.37 ±13.9 (25-88)	0.225
**Gender (%)**			
Male	29 (73)	68 (78)	0.485
Female	11 (27)	19 (22)	
**Smoking status (%)**			
Never	12 (30)	20 (23)	0.398
Current/former	28 (70)	67 (77)	
**Alcohol status (%)**			
Never	10(25)	13 (15)	0.172
Current/former	30(75)	74 (85)	
**pT stage (%)**			
T1-2	9 (23)	27 (31)	0.322
T3-4	31 (77)	60 (69)	
**pN stage (%)**			
N0-1	16 (40)	29 (33)	0.466
T2-3	24 (60)	58 (67)	
**Histological differentiation (%)**			
Well	31 (77)	50 (58)	0.029
Moderate/poor	9 (23)	37 (42)	
**Extranodal spread of lymph node metastasis (%)**			
Absent	30 (75)	66 (76)	0.916
Present	10 (25)	21 (24)	
**Positive margin (%)**			
Absent	34 (85)	73 (84)	0.875
Present	6 (15)	14 (16)	
**Vascular invasion (%)**			
Absent	12 (30)	12 (14)	0.030
Present	28 (70)	75 (86)	
**Lymphatic invasion (%)**			
Absent	33 (83)	68 (78)	0.573
Present	7 (18)	19 (22)	
**Perineural invasion (%)**			
Absent	15 (27)	44 (51)	0.170
Present	25 (63)	43 (49)	

NOTE: The t-test was used for continuous variables and the Pearson's chi-square test for categorical variables. †Continuous variables. CK4, cytokeratin4; PORT, postoperative radiotherapy; POCRT, postoperative chemoradiotherapy.

Supplementary Table 5: Datasets used in the validation study

Dataset	Number of samples	Nature of samples	Prognostic data	Paper publication/ author
GSE31056	23	TSCC: 23<br>Stage III/IV: 9)	Relapse-free survival<br>RFS)	*BMC Cancer* 2011,11:437/<br>Patricia P Reis et. al [33]
GSE42743	49	OSCC: 49<br>Stage III/IV: 49)	Disease-specific survival<br>(DSS)	*Clin Cancer Res*.<br>2013,1;19(5):1197-203./<br>Pawadee L et. al [24]
GSE41613	51	OSCC: 51<br>Stage III/IV: 51)	Disease-specific survival<br>DSS)	

NOTE: The primary expression microarray data are available at Gene Expression Omnibus (http://www.ncbi.nlm.nih.gov/geo/; NCBI). TSCC, tongue squamous cell carcinoma; OSCC, oral squamous cell carcinoma.

Supplementary Table 6: List of primer sequences used for RT-qPCR analysis

Genes	Primer sequences
KRT4	F: CCATCAACCAGAGCTTGCTCAC<br>R: TCCATTTGGTCTCCAGGACCTTA
GAPDH	F: GCACCGTCAAGGCTGAGAAC<br>R: TGGTGAAGACGCCAGTGGA

NOTE: F; Forward primer, R; reverse primer.
