# Supplementary material for: Gene expression profiling to predict recurrence of advanced squamous cell carcinoma of the tongue: discovery and external validation
Source: Oncotarget. 2017 Jun 27;8(37):61786–99. doi: 10.18632/oncotarget.18692 (PMC5617464; doi:10.18632/oncotarget.18692)
Supplement: Supplementary file 2 [file oncotarget-08-61786-s002.docx]

**Supplementary Table 1: Clinicopathologic characteristics of patients in the discovery study classified according to unsupervised hierarchical clustering**

| **Variable** | **Cluster A**  (n=10) | **Cluster B**  (n=16) | ***P*** **value** |
| --- | --- | --- | --- |
| **Age**^†^ Mean ±SD (range) | 58.4 ±13.0 (47-85) | 60.9 ±14.3 (37-80) | 0.21 |
| **Gender** (%)  Male  Female | 1 (10)  9 (90) | 9 (56)  7 (44) | 0.07 |
| **Smoking status** (%)  Never  Current/former | 2 (20)  8 (80) | 7 (44)  9 (56) | 0.216 |
| **Alcohol status** (%)  Never  Current/former | 3 (30)  7 (70) | 7 (44)  9 (56) | 0.483 |
| **Surgery** (%)  Partial resection  Hemi glossectomy  Subtotal glossectomy  Total glossectomy | 1 (10)  3 (30)  5 (50)  1 (10) | 2 (13)  1 (6)  12 (75)  1 (6) | 0.493 |
| **Treatment form** (%)  Surgery alone  Surgery + PORT  Surgery + POCRT | 9 (90)  0 (0)  1 (10) | 13 (81)  2 (13)  1 (6) | 0.614 |
| **pT stag**e (%)  T1-2  T3-4 | 1 (10)  9 (90) | 2 (13)  14 (87) | 0.846 |
| **pN stage** (%)  N0-1  T2-3 | 2 (20)  8 (80) | 8 (50)  8 (50) | 0.126 |
| **UICC pStage** (%)  Stage III  Stage IVa  Stage IVb | 0 (0)  10 (100)  0 (0) | 2 (13)  14 (87)  0 (0) | 0.245 |
| **Histological differentiation** (%)  Well  Moderate/poor | 8 (80)  2 (20) | 12 (75)  4 (25) | 0.768 |
| **Extranodal spread of**  **lymph node metastasis** (%)  Absent  Present | 8 (80)  2 (20) | 16 (100)  0 (0) | 0.063 |
| **Positive margin** (%)  Absent  Present | 10 (100)  0 (0) | 15 (94)  1 (6) | 0.420 |
| **Vascular invasion** (%)  Absent  Present | 1 (10)  9 (90) | 3 (12)  13 (88) | 0.547 |
| **Lymphatic invasion** (%)  Absent  Present | 9 (90)  1 (10) | 12 (75)  4 (25) | 0.345 |
| **Perineural invasion** (%)  Absent  Present | 6 (60)  4 (40) | 9 (56)  7 (44) | 0.851 |
| **Alcohol status**  Never  Current/former | 3(30)  7(70) | 7(43.7)  9(53.3) | 0.483 |

NOTE: The t-test was used for continuous variables and the Pearson test or Kruskal-Wallis test for categorical variables. ^†^Continuous variables. UICC, Union for International Cancer Control; PORT, postoperative radiotherapy; POCRT, postoperative chemoradiotherapy.
