# Supplementary material for: Gene expression profiling to predict recurrence of advanced squamous cell carcinoma of the tongue: discovery and external validation
Source: Oncotarget. 2017 Jun 27;8(37):61786–99. doi: 10.18632/oncotarget.18692 (PMC5617464; doi:10.18632/oncotarget.18692)
Supplement: Supplementary file 3 [file oncotarget-08-61786-s003.docx]

**Supplementary Table 2: Functional Enrichment Analyses in the discovery cohort**

**<Annotations enriched in cluster A>**

| **Annotation** | **Size** | **Enrichment Score** | ***P* value** |
| --- | --- | --- | --- |
| KEGG_GLYCOSAMINOGLYCAN_  BIOSYNTHESIS_CHONDROITIN_SULFATE | 19 | 0.80480814 | <0.001 |
| PID_ILK_PATHWAY | 44 | 0.54259163 | <0.001 |
| PID_P73PATHWAY | 76 | 0.404181 | <0.001 |
| KEGG_SMALL_CELL_LUNG_CANCER | 80 | 0.48771736 | 0.002132196 |
| REACTOME_ASPARAGINE_  N_LINKED_GLYCOSYLATION | 76 | 0.4993559 | 0.002145923 |
| PID_SYNDECAN_4_PATHWAY | 28 | 0.6667868 | 0.002197802 |
| REACTOME_CHONDROITIN_SULFATE_  DERMATAN_SULFATE_METABOLISM | 41 | 0.6712772 | 0.002217295 |
| PID_SYNDECAN_1_PATHWAY | 44 | 0.6352256 | 0.008316008 |
| PID_FAK_PATHWAY | 57 | 0.49428686 | 0.008510638 |
| PID_AVB3_INTEGRIN_PATHWAY | 72 | 0.5581143 | 0.010683761 |
| REACTOME_A_TETRASACCHARIDE_LINKER_  SEQUENCE_IS_REQUIRED_FOR_GAG_SYNTHESIS | 20 | 0.69387394 | 0.010893246 |
| PID_INTEGRIN1_PATHWAY | 62 | 0.69111717 | 0.010989011 |
| KEGG_REGULATION_OF_ACTIN_CYTOSKELETON | 181 | 0.36083895 | 0.011185682 |
| REACTOME_GLYCOSAMINOGLYCAN_  METABOLISM | 91 | 0.4705473 | 0.011235955 |
| REACTOME_PHOSPHORYLATION_OF_THE_APC_C | 16 | 0.6557887 | 0.012269938 |
| REACTOME_HEPARAN_SULFATE_  HEPARIN_HS_GAG_METABOLISM | 42 | 0.4917991 | 0.013452915 |
| KEGG_N_GLYCAN_BIOSYNTHESIS | 45 | 0.5044731 | 0.014613778 |
| KEGG_FOCAL_ADHESION | 186 | 0.48211884 | 0.015250545 |
| REACTOME_INHIBITION_OF_THE_PROTEOLYTIC_ACTIVITY_OF_APC_C_REQUIRED_FOR_THE_  ONSET_OF_ANAPHASE_BY_MITOTIC_SPINDLE_  CHECKPOINT_COMPONENTS | 17 | 0.6554924 | 0.015904572 |
| REACTOME_PROTEIN_FOLDING | 45 | 0.4925555 | 0.01632653 |
| REACTOME_TRANSPORT_TO_THE_GOLGI_  AND_SUBSEQUENT_MODIFICATION | 31 | 0.4966946 | 0.016842104 |
| KEGG_GLYCOSAMINOGLYCAN_  BIOSYNTHESIS_HEPARAN_SULFATE | 20 | 0.5356362 | 0.017857144 |
| PID_A6B1_A6B4_INTEGRIN_PATHWAY | 43 | 0.5349235 | 0.0186722 |
| PID_SYNDECAN_2_PATHWAY | 30 | 0.55048215 | 0.018947368 |
| KEGG_ECM_RECEPTOR_INTERACTION | 75 | 0.6222889 | 0.019067796 |
| REACTOME_CLASS_I_MHC_ MEDIATED_  ANTIGEN_PROCESSING_PRESENTATION | 220 | 0.44864708 | 0.019880716 |
| PID_P75_NTR_PATHWAY | 64 | 0.41281247 | 0.021598272 |
| PID_INTEGRIN3_PATHWAY | 38 | 0.63429314 | 0.023305085 |
| REACTOME_METABOLISM_OF_CARBOHYDRATES | 187 | 0.3080636 | 0.023655914 |
| REACTOME_COLLAGEN_FORMATION | 55 | 0.6707199 | 0.023758098 |
| KEGG_PATHWAYS_IN_CANCER | 284 | 0.32506567 | 0.02494331 |
| REACTOME_REGULATION_OF_  MITOTIC_CELL_CYCLE | 74 | 0.6546296 | 0.025896415 |
| REACTOME_APC_C_CDH1_MEDIATED_DEGRADATION_OF_CDC20_AND_OTHER_APC_C_CDH1_TARGETED_PROTEINS_  IN_LATE_MITOSIS_EARLY_G1 | 61 | 0.66938496 | 0.029239766 |
| REACTOME_APC_C_CDC20_MEDIATED_  DEGRADATION_OF_MITOTIC_PROTEINS | 62 | 0.6664224 | 0.02952756 |
| REACTOME_APC_C_CDC20_MEDIATED_  DEGRADATION_OF_CYCLIN_B | 18 | 0.58998895 | 0.030364372 |
| REACTOME_AUTODEGRADATION_OF_  CDH1_BY_CDH1_APC_C | 54 | 0.65594035 | 0.031434186 |
| REACTOME_ANTIGEN_PROCESSING_  UBIQUITINATION_PROTEASOME_DEGRADATION | 184 | 0.4372435 | 0.03245436 |
| PID_REELIN_PATHWAY | 26 | 0.5294099 | 0.033542976 |
| PID_LYMPH_ANGIOGENESIS_PATHWAY | 24 | 0.55921423 | 0.033755273 |
| KEGG_PROGESTERONE_MEDIATED_OOCYTE_MATURATION | 78 | 0.36546168 | 0.03411514 |
| ST_INTEGRIN_SIGNALING_PATHWAY | 77 | 0.43703738 | 0.034408603 |
| REACTOME_RECYCLING_PATHWAY_OF_L1 | 25 | 0.4954978 | 0.034408603 |
| NABA_COLLAGENS | 39 | 0.66829604 | 0.03470716 |
| REACTOME_HS_GAG_BIOSYNTHESIS | 26 | 0.48035046 | 0.034802783 |
| BIOCARTA_ARAP_PATHWAY | 17 | 0.5862222 | 0.03655914 |
| REACTOME_ACTIVATED_NOTCH1_  TRANSMITS_SIGNAL_TO_THE_NUCLEUS | 24 | 0.5063512 | 0.037815128 |
| REACTOME_G_BETA_GAMMA_SIGNALLING_  THROUGH_PLC_BETA | 16 | 0.52238935 | 0.037861917 |
| REACTOME_EXTRACELLULAR_MATRIX_ORGANIZATION | 73 | 0.6403455 | 0.03887689 |
| BIOCARTA_AGR_PATHWAY | 31 | 0.5021146 | 0.0397351 |
| PID_NOTCH_PATHWAY | 54 | 0.41482416 | 0.041394334 |
| PID_INTEGRIN_CS_PATHWAY | 24 | 0.5701948 | 0.041753653 |
| REACTOME_APC_CDC20_MEDIATED_  DEGRADATION_OF_NEK2A | 20 | 0.5714725 | 0.04183267 |
| REACTOME_AMINO_ACID_TRANSPORT_  ACROSS_THE_PLASMA_MEMBRANE | 22 | 0.5082885 | 0.04185022 |
| REACTOME_L1CAM_INTERACTIONS | 74 | 0.38921252 | 0.04385965 |
| REACTOME_DEVELOPMENTAL_BIOLOGY | 324 | 0.30382577 | 0.044705883 |
| REACTOME_CONVERSION_FROM_APC_C_  CDC20_TO_APC_C_CDH1_IN_LATE_ANAPHASE | 15 | 0.5809125 | 0.044806518 |
| NABA_BASEMENT_MEMBRANES | 34 | 0.67302805 | 0.04585153 |
| REACTOME_AXON_GUIDANCE | 214 | 0.3714268 | 0.046153847 |
| BIOCARTA_UCALPAIN_PATHWAY | 17 | 0.605791 | 0.047826085 |
| REACTOME_ANTIGEN_PROCESSING_  CROSS_PRESENTATION | 69 | 0.58527297 | 0.048 |
| PID_INTEGRIN_A4B1_PATHWAY | 32 | 0.46928847 | 0.04842105 |
| KEGG_PRION_DISEASES | 27 | 0.5024535 | 0.048672568 |
| PID_UPA_UPAR_PATHWAY | 37 | 0.4862634 | 0.04899777 |
| REACTOME_CHONDROITIN_SULFATE_BIOSYNTHESIS | 16 | 0.676161 | 0.0494382 |

**< Annotations enriched in cluster B>**

| **Annotation** | **Size** | **Enrichment Score** | ***P* value** |
| --- | --- | --- | --- |
| KEGG_ARACHIDONIC_ACID_METABOLISM | 41 | -0.67600685 | <0.001 |
| KEGG_LINOLEIC_ACID_METABOLISM | 17 | -0.80811274 | <0.001 |
| REACTOME_BIOLOGICAL_OXIDATIONS | 90 | -0.60338664 | <0.001 |
| KEGG_DRUG_METABOLISM_CYTOCHROME_P450 | 49 | -0.6868605 | <0.001 |
| KEGG_METABOLISM_OF_XENOBIOTICS_  BY_CYTOCHROME_P450 | 44 | -0.69334203 | <0.001 |
| REACTOME_CYTOCHROME_P450_ARRANGED_  BY_SUBSTRATE_TYPE | 32 | -0.65682995 | <0.001 |
| REACTOME_PHASE1_  FUNCTIONALIZATION_OF_COMPOUNDS | 50 | -0.6385077 | <0.001 |
| KEGG_STEROID_HORMONE_BIOSYNTHESIS | 23 | -0.63662684 | 0.001897533 |
| REACTOME_PHASE_II_CONJUGATION | 41 | -0.5528568 | 0.001996008 |
| KEGG_RETINOL_METABOLISM | 35 | -0.5669518 | 0.003759399 |
| PID_MAPK_TRK_PATHWAY | 31 | -0.5025934 | 0.003921569 |
| REACTOME_ACYL_CHAIN_REMODELLING_OF_PE | 15 | -0.6784692 | 0.005703422 |
| REACTOME_CIRCADIAN_CLOCK | 48 | -0.42525262 | 0.015748031 |
| REACTOME_TIGHT_JUNCTION_INTERACTIONS | 23 | -0.6909919 | 0.016260162 |
| REACTOME_CTNNB1_  PHOSPHORYLATION_CASCADE | 15 | -0.58138317 | 0.020576132 |
| KEGG_VALINE_LEUCINE_AND_  ISOLEUCINE_DEGRADATION | 44 | -0.5764111 | 0.023622047 |
| REACTOME_RORA_ACTIVATES_  CIRCADIAN_EXPRESSION | 23 | -0.48554552 | 0.026639344 |
| REACTOME_GLUTATHIONE_CONJUGATION | 18 | -0.67836213 | 0.027184466 |
| REACTOME_BILE_ACID_AND_  BILE_SALT_METABOLISM | 17 | -0.59512067 | 0.027777778 |
| REACTOME_TIE2_SIGNALING | 16 | -0.51192164 | 0.036893204 |
| REACTOME_BMAL1_CLOCK_NPAS2_  ACTIVATES_CIRCADIAN_EXPRESSION | 33 | -0.44116628 | 0.04255319 |
| KEGG_PROPANOATE_METABOLISM | 29 | -0.5849826 | 0.044265594 |
| REACTOME_CIRCADIAN_REPRESSION_  OF_EXPRESSION_BY_REV_ERBA | 21 | -0.4609056 | 0.045725647 |
| REACTOME_PI3K_EVENTS_IN_ERBB2_SIGNALING | 40 | -0.4490756 | 0.046728972 |
| REACTOME_BRANCHED_CHAIN_  AMINO_ACID_CATABOLISM | 17 | -0.6482599 | 0.049701788 |
| REACTOME_ION_TRANSPORT_BY_  P_TYPE_ATPASES | 28 | -0.47092518 | 0.04990403 |

NOTE: Size means number of human genes with an ontology annotation of this term. Enrichment scores are used to benchmark expression levels in cluster A compared to cluster B (positive value means that the pathway is enriched in cluster A). The absolute values show the magnification.
